# Electrochemical Sensor of Ciprofloxacin on Screen-Printed Electrode Modified with Boron-Doped Diamond Nanoparticles and Nickel Oxide Nanoparticles Biosynthesized Using *Spatholobus littoralis Hassk.* Root Extract

**DOI:** 10.3390/bios16030148

**Published:** 2026-03-03

**Authors:** Laurencia Gabrielle Sutanto, Prastika Krisma Jiwanti, Mirza Ardella Saputra, Mai Tomisaki, Nurul Mutmainah Diah Oktaviani, Widiastuti Setyaningsih, Yasuaki Einaga, Tahta Amrillah, Ilma Amalina, Wan Jeffrey Basirun, Qonita Kurnia Anjani

**Affiliations:** 1 Nanotechnology Engineering, Faculty of Advanced Technology and Multidiscipline, Universitas Airlangga, Surabaya 60115, Indonesiamirza.ardella@ftmm.unair.ac.id (M.A.S.); tahta.amrillah@ftmm.unair.ac.id (T.A.);; 2 Airlangga Functional Nanomaterials Research Group, Faculty of Advanced Technology and Multidiscipline, Universitas Airlangga, Surabaya 60115, Indonesia; 3 International Institute for Carbon-Neutral Energy Research (WPI-I2CNER), Kyushu University, 744 Motooka, Nishi-ku, Fukuoka 819-0395, Japan; tomisaki.mai.350@m.kyushu-u.ac.jp; 4 School of Industrial Engineering, Telkom University, Telkom University Landmark Tower Lt. 18, Jl. Telekomunikasi 1, Terusan Buahbatu—Bojongsoang, Jawa Barat 40257, Indonesia; 5 Department of Food and Agricultural Product Technology, Faculty of Agricultural Technology, Universitas Gadjah Mada, Jalan Flora No 1, Bulaksumur, Depok, Sleman, Yogyakarta 55281, Indonesia; widiastuti.setyaningsih@ugm.ac.id; 6 Department of Chemistry, Keio University, 3-14-1 Hiyoshi, Yokohama 223-8522, Japan; einaga@keio.jp; 7 Department of Chemistry, Faculty of Science, University Malaya, Kuala Lumpur 50603, Malaysia; jeff@um.edu.my; 8 School of Pharmacy, Medical Biology Centre, Queen’s University Belfast, 97 Lisburn Road, Belfast BT9 7BL, UK; qonita.anjani@qub.ac.uk

**Keywords:** ciprofloxacin, nickel oxide nanoparticles, screen-printed electrode, boron-doped diamond nanoparticles, good health and well-being

## Abstract

Ciprofloxacin (CIP) is an antibiotic that is widely used in humans and animals. However, the compound has been detected in animal-derived products and the environment due to its extensive use, causing serious concern for public health and environmental safety. The issue raises the urgent need to develop innovative techniques to monitor CIP. Therefore, this study aims to develop a simple and sensitive CIP sensor called the boron-doped diamond nanoparticle-modified screen-printed electrode (BDD NPs/SPE) and the nickel oxide nanoparticle-modified BDD NPs/SPE (NiO NPs/BDD NPs/SPE). NiO NPs were synthesized via green synthesis using *Spatholobus littoralis Hassk.* root extract as the reducing agent. The formation and characteristics of NiO NPs were then confirmed through a UV-Vis spectrophotometer, XRD, PSA, FT-IR, and XPS. The successful modification of SPE was confirmed through SEM-EDX, followed by measurements using square-wave voltammetry. The results showed that the modified SPE could detect CIP over a concentration range of 0.1–100 µM and produced a low detection limit of 0.109 µM for BDD NPs/SPE and 0.054 µM for NiO NPs/BDD NPs/SPE. The proposed method was successfully applied to the determination of CIP in commercial tablets, milk, and human urine, with a satisfactory % recovery from 95 to 100%. The current study successfully developed a simple yet highly sensitive sensor that enabled robust, reliable, and efficient detection of CIP, showing its strong potential for practical applications.

## 1. Introduction

Ciprofloxacin (CIP) is one of the fluoroquinolone antibiotics that are most widely used to treat various bacterial infections of both Gram-positive and Gram-negative bacteria, including *Escherichia coli*, *Salmonella*, *Shigella*, and *Pseudomonas aeruginosa* [1,2]. CIP works by inhibiting the activity of the DNA gyrase and topoisomerase IV enzymes that play a role in the process of DNA replication and bacterial cell division [3]. The drug has also been widely used to treat various infections, including those of the urinary tract, respiratory tract, digestive tract, skin, soft tissue, bones, joints, and anthrax [4].

According to previous studies, CIP is one of the antibiotics that are most commonly used to prevent and treat bacterial infections in livestock and pets [5]. Widespread use of CIP in livestock can lead to the contamination of animal products. Several studies have found its contamination in various common animal products, including milk, chicken meat, and beef [6,7,8]. CIP contamination can lead to severe side effects to human health when consumed, including psychosis, seizures, tendinitis, myopathy, and aortic aneurysm [9]. In addition, its widespread use can pollute water and soil [10,11], leading to the development of antibiotic-resistant bacteria that threaten global health [5]. This indicates that it is important to develop methods for the monitoring of CIP.

The determination of CIP has been carried out using various methods, including High-Performance Liquid Chromatography (HPLC), as reported by Bosma et al. (2020) [6], and fluorometry, as reported by Lu et al. (2020) [12]. These methods have high sensitivity and allow for the analysis of the compound at low concentrations. However, both have several shortcomings, such as being expensive, time-consuming, and having complicated operations. In recent decades, electrochemical sensors have become one of the most promising instruments for sensing because of their simple, quick, and cost-effective nature [13].

Screen-printed electrodes (SPEs) have also become widely used in electrochemical analysis due to their efficiency, affordability, and portability. These electrodes are commonly modified with functional materials to improve sensitivity and analytical performance. Among various modifiers, carbon-based materials are attractive because of their high surface area and chemical inertness, while metal-based nanoparticles are valued for their catalytic properties that enhance electrode reactivity [14,15]. For example, Jiwanti et al. (2024) modified SPEs with reduced graphene oxide (rGO) and SnO_2_ to develop a CIP sensor with a limit of detection (LOD) of 2.03 μM [16].

As a SPE modifier, boron-doped diamond nanoparticles (BDD NPs) have gained considerable attention for electrochemical sensor applications due to their high surface area, excellent chemical and physical stability, and biocompatibility [17]. These particles have been examined for antibiotic detection, such as levofloxacin, achieving a LOD of 2.24 μM for [18]. However, while BDD NPs provide a stable and low-background conductive platform, further electrocatalytic activity enhancement is often required to improve charge transfer efficiency and sensitivity. In this context, nickel oxide nanoparticles (NiO NPs) emerge as a promising electrocatalytic material due to their semiconducting nature, better conductivity, and chemical stability [19,20]. The addition of NiO NPs to a sensor can improve overall sensor capability.

NiO NP synthesis can be carried out by various methods, including sol–gel, coprecipitation, and hydrothermal methods [21]. Compared to these methods, green synthesis has garnered attention due to its non-toxic, low energy consumption, cost-effective, and environmentally friendly characteristics [22]. The use of green-synthesized NiO NPs as a modifier for an electrochemical sensor resulted in a great effect. A study conducted by Youcef et al. (2022) found that a glassy carbon electrode (GCE) modified with NiO NPs achieved a LOD of 6.15 µM for glucose detection [23]. Green synthesis involves natural materials as reducing agents and stabilizers of nanoparticles, such as plant extracts, bacteria, fungi, and yeast [24]. Bajakah root (*Spatholobus littoralis Hassk.*) is a native Indonesian herbal plant from Central Kalimantan with abundant availability and is rich in bioactive compounds, including phenols, flavonoids, and terpenoids. The plant was explored in this study as a sustainable reducing and stabilizing agent for NiO NP synthesis [25].

In the current study, NiO NPs were synthesized using *S. littoralis Hassk.* root extract, and BDD NPs were employed to modify SPEs via a simple drop-casting method to develop a sensitive and selective electrochemical sensor for CIP detection. The electrochemical behavior and analytical performance were evaluated using cyclic voltammetry (CV) and square-wave voltammetry (SWV), respectively. The proposed sensor was then applied to a commercial tablet, milk, and human urine to assess real-sample application. Method validation, including linearity, sensitivity, LOD, LOQ, accuracy, and reproducibility were investigated.

## 2. Materials and Methods

### 2.1. Materials and Reagents

The materials and reagents used in this study included ciprofloxacin (≥98%) (Sigma Aldrich, Darmstadt, Germany), nickel chloride (NiCl_2_, ≥98%) (Sigma Aldrich, Darmstadt, Germany), boron-doped diamond nanoparticle powder 0–250 nm (Boromond, Changsha, China), screen-printed electrodes (Tailkuke, China), Bajakah root (*S. littoralis Hassk.*), sulfuric acid (H_2_SO_4_, ≥98%) (Sigma Aldrich, Darmstadt, Germany), disodium hydrogen phosphate (Na_2_HPO_4_, ≥99%) (Millipore, Darmstadt, Germany), sodium dihydrogen phosphate (NaH_2_PO_4_, ≥99%) (Millipore, Darmstadt, Germany), D-glucose (Nitra Kimia, Yogyakarta, Indonesia), LEV (≥98%) (Sigma Aldrich, Darmstadt, Germany), ofloxacin (OFLO) (≥98%) (Sigma Aldrich, Darmstadt, Germany), ascorbic acid (Nitra Kimia, Yogyakarta, Indonesia), sodium chloride (NaCl) (Merck, Darmstadt, Germany) (≥99.5%), urea (Nitra Kimia, Yogyakarta, Indonesia), ethanol absolute (≥99.9%) (Supelco, Darmstadt, Germany), methanol (≥99.9%) (Supelco, Darmstadt, Germany), distilled water (Sumber Ilmiah Persada, Surabaya, Indonesia), CIP commercial tablets, milk samples, and urine. The SPEs (working electrode ⌀ = 0.5 cm) with a three-electrode configuration were purchased from Poten Technology Co., Ltd., Weihai, China.

### 2.2. Extraction of S. littoralis Hassk

Roots of the plant (*S. littoralis Hassk.*) were rinsed with distilled water and dried at room temperature for 1 day. The dried roots were ground into coarse powder, then weighed to 10 g and boiled in 100 mL of 40% methanol solution at 80 °C for 30 min. The mixture was cooled and filtered using a vacuum filtration pump to separate the supernatant and filtrate. The resulting supernatant was stored in a refrigerator at 4 °C for further procedures.

### 2.3. Synthesis of NiO NPs

The extract solution of *S. littoralis Hassk.* was added into 100 mL of 0.1 M NiCl_2_ solution (50% *v*/*v*). The mixture was stirred constantly until homogeneous, then a 5 M NaOH solution was added until the pH of the solution reached 12. Furthermore, the homogeneous solution was stirred and heated at 60 °C for 60 min. The formation of NiO NPs was indicated by a change in the color of the solution from cyan to dark brown. The precipitate of NiO NPs was separated using centrifugation at 4000 rpm for 15 min and washed with absolute ethanol 3 times to remove impurities. The NiO NP powder was dried in an oven at 80 °C for 12 h and then stored for characterization procedures.

### 2.4. Characterization of NiO NPs

The formation of NiO NPs was confirmed by UV-Vis spectrophotometer (Thermoscientific Orion Aquamate, Thermo Fisher Scientific Inc., Waltham, MA, USA) analysis at a wavelength of 200–700 nm. The particle size of NiO NPs was measured using dynamic light scattering (DLS) (Delsa Nano, Beckman Coulter, Inc., Brea, CA, USA). Furthermore, the morphology and composition of NiO NPs were observed using a scanning electron microscope–energy dispersive X-ray (SEM-EDX) (JSM-7900F, JEOL Ltd., Tokyo, Japan). X-ray diffractometer (XRD) (Rigaku MiniFlex 600-C, Rigaku, Tokyo, Japan) characterization was performed on NiO NP powder that had been calcined at 400 °C for 2 h to determine the level of crystallinity, crystal phase, and crystallite size of NiO NPs. The bioactive content in the *S. littoralis Hassk.* extract that contributed to the reduction process of NiO NPs was analyzed by Fourier transform infrared (FT-IR) spectroscopy (Shimadzu Tracer-100, Shimadzu Corporation, Kyoto, Japan). The electronic properties and chemical bonds of NiO NPs were characterized using an X-ray photoelectron spectrometer (XPS) (PHI5000VersaProbe II, ULVAC-PHI, Kanagawa, Japan).

### 2.5. Fabrication of Modified Electrode

Modification of the SPEs with BDD NPs and NiO NPs was carried out using the drop-casting technique as developed by Jiwanti et al. (2023) [26]. Preparation of the BDD NP conductive ink was conducted by dissolving 10 mg of the BDD NPs powder into 0.5 mL of a 30% ethanol solution. Furthermore, 4 µL of the BDD NP conductive ink was dropped onto the SPE working electrode (BDD NPs/SPE) and then dried at 50 °C for 5 min. The modification process was continued by dropping 4 µL of the biosynthesized NiO NP solution onto the BDD NPs/SPE working electrode, with the same drying method as before, annotated as NiO NPs/BDD NPs/SPE. Furthermore, both electrodes were characterized using SEM-EDX to observe the success of the modification.

### 2.6. Electrochemical Measurement and Determination of Real Sample

All electrochemical measurements were performed using an Emstat3+ blue potentiostat from Palmsens, assisted by PSTrace Software 5.8. CV measurements were carried out to determine the electrochemical behavior of CIP. In this study, CIP detection was carried out using the SWV, with the following parameters: a potential range of 0 to 1.2 V (vs. Ag/AgCl), a frequency of 50 Hz, an amplitude of 0.05 V, and a potential step of 0.012 V. Electrochemical validation included signal per background (S/B) ratio, linearity, precision, accuracy, selectivity, and real sample measurement. Selectivity tests were carried out by adding interfering compounds, including 100 µM D-glucose, 60 µM LEV, 60 µM OFLO, 60 µM ascorbic acid, 4 µM NaCl, and 10 µM urea. CIP in real samples was measured in human urine, cow’s milk, and commercial tablets. Human urine was obtained from a healthy volunteer in the morning before the measurement. Cow’s milk and commercial tablets were purchased from a local supermarket and pharmacy, respectively. The commercial tablets were dissolved and diluted to a concentration of 100 µM, and the cow’s milk and human urine were spiked with 100 µM CIP in 0.1 M phosphate buffer solution (pH 7.0), which were selected to represent near-neutral conditions relevant to biological and dairy samples.

## 3. Results and Discussion

### 3.1. Characterization of NiO NPs

**Figure 1:**
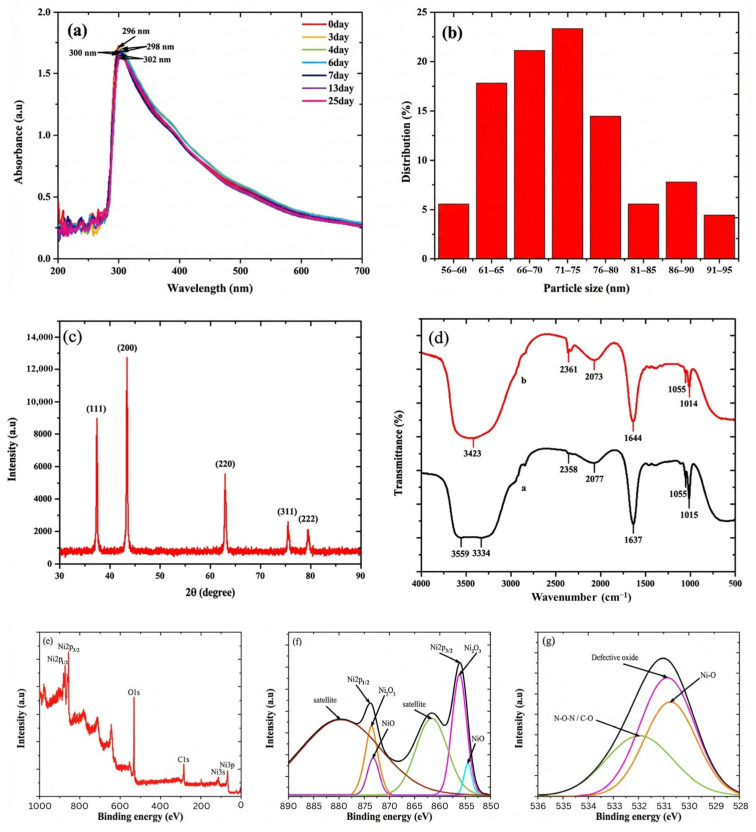
Characterization of NiO NPs. (**a**) UV-Vis spectrum, (**b**) particle size distribution, (**c**) XRD spectrum, (**d**) FT-IR, (**e**) XPS wide scan, (**f**) XPS narrow scan of Ni2p, and (**g**) XPS narrow scan of O1s.

The formation of NiO NPs and their stability were monitored using UV-Vis spectroscopy. The specific maximum absorption peak of the NiO NPs ranged from 230 to 400 nm, which was formed due to the occurrence of surface plasmon resonance (SPR) [27]. As shown in Figure 1a, the maximum absorption peak of the NiO NPs was located at a wavelength of around 296 nm. The stability of the NiO NPs was tested during 25 days of storage in a refrigerator at 4 °C. A decrease in absorbance of 8.21% and a shift in wavelength to 302 nm were observed from the stored sample. The measurement results for 25 days did not show any significant difference, proving that NiO NPs have good stability. The NiO NP particle size showed a size range of 56 to 95 nm, with an average size of 73 nm (Figure 1b). Furthermore, the NiO NPs showed a narrow and uniform size distribution, as evidenced by the polydispersity index (PI) value of 0.261 [28].

The successful formation of NiO NPs was confirmed through XRD characterization. In this study, the biosynthetic NiO NPs had high crystallinity as indicated by the formation of five sharp diffraction peaks at 37.35°, 43.38°, 62.95°, 75.47°, and 79.46°, which showed the crystallographic reflections of the NiO NPs (111), (200), (220), (311), and (222) planes, respectively (Figure 1c). The formed Bragg reflection peaks indicated that the crystal phase of the NiO NPs was face-centered cubic (FCC) [29]. Through the Debye–Scherrer equation, the crystallite size of the NiO NPs was 0.69 nm. The crystallite size was influenced by the nucleation rate and growth rate. When the growth rate is higher than the nucleation rate, the resulting crystallite size tends to be larger, and when the nucleation rate is higher than the growth rate, the resulting crystallite size will be smaller [30]. Smaller crystal sizes caused changes in atomic transport and the electron energy spectrum, which could increase catalytic activity, ensuring that the modification of the SPE with the NiO NPs improved sensor performance [31].

**Figure 2:**
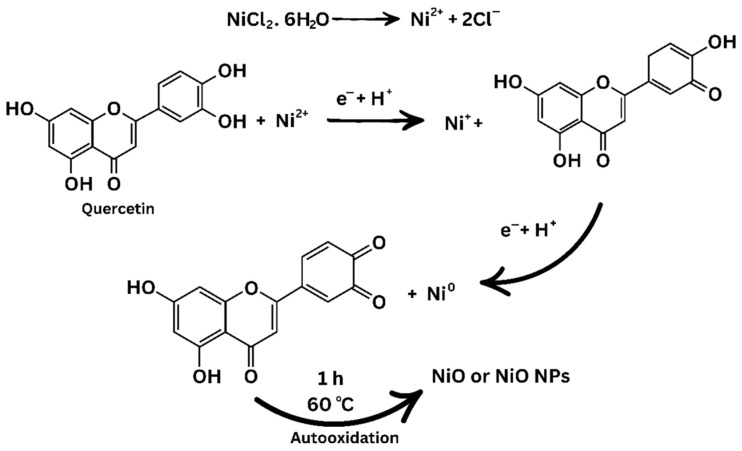
Reduction mechanism of NiO NPs.

The involvement of bioactive ingredients in the *S. littoralis Hassk.* extract in the synthesis of NiO NPs was investigated through FT-IR analysis. The FT-IR spectrum of the *S. littoralis Hassk.* extract showed seven peaks corresponding to O-H stretching of methanol (3559 cm^−1^) [32], N-H stretching of secondary aliphatic amine (3334 cm^−1^) [33], C-H stretching of aliphatic compounds (2358 cm^−1^) [34], H−C=O stretching (2077 cm^−1^), C=O stretching (1637 cm^−1^) [35], C−O stretching (1055 cm^−1^) [36], and C−O stretching (1015 cm^−1^) [36]. Meanwhile, the FT-IR spectrum of the NiO NPs showed six peaks corresponding to O-H stretching (3423 cm^−1^) [37], C-H stretching of aliphatic compounds (2361 cm^−1^) [34], H−C=O stretching (2073 cm^−1^) [34], C=O stretching (1644 cm^−1^) [35], C−O stretching (1055 cm^−1^) [36], and C−O stretching (1014 cm^−1^) [36] (Figure 1d). The disappearance of the peak at 3334 cm^−1^ and the decrease in the intensity of the peaks at 2077, 1637, and 1015 cm^−1^ [38,39,40] indicate that the formation mechanism of the NiO NPs involved the reduction reaction of Ni^2+^ ions and the oxidation of compounds that act as a reducing agent (Figure 2).

The electronic properties and chemical bonds of the biosynthesized NiO NPs were analyzed using XPS. The XPS spectrum confirmed that NiO NPs were successfully formed. In this study, six sharp peaks were observed, which were the spectra of Ni2p_1/3_ (874 eV), Ni2p_3/2_ (856 eV), O1s (531 eV), C1s (286 eV), Ni3s (118 eV), and Ni3p (70 ev) (Figure 1e). The deconvolution of the Ni2p spectrum showed six peaks, which corresponded to Ni^2+^ from the Ni-O bond (855 and 872 eV) [41], Ni^3+^ from Ni_2_O_3_ (856 and 874 eV) [42], and satellite peaks belonging to Ni2p_3/2_ and Ni2p_1/2_ (861 and 880 eV) [43] (Figure 1f). Meanwhile, the deconvolution of the XPS O1s spectrum showed three peaks, belonging to O^2−^ (529 eV) [44], a defective oxide peak (531 eV) [45], and H-O-H or C-O (532 eV) [45] (Figure 1g).

**Figure 3:**
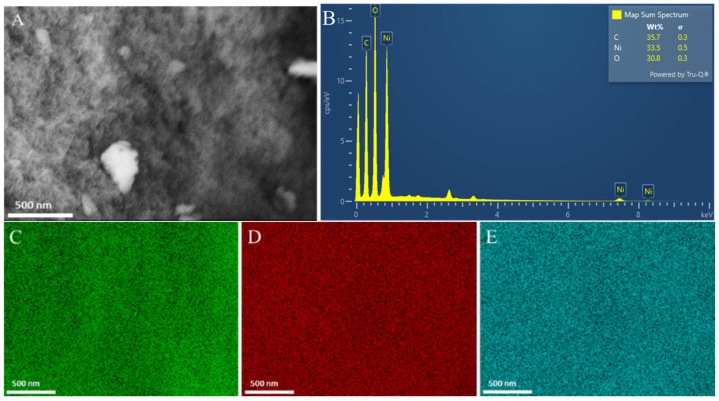
(**A**) SEM image (magnified 50,000×) of NiO NPs, (**B**) EDX spectrum of NiO NPs, and (**C**–**E**) elemental mapping of carbon, nickel, and oxygen.

SEM characterization was performed to investigate the surface morphology of the NiO NPs. Furthermore, the SEM images showed that the NiO NPs had a spherical shape (Figure 3A). The composition and purity of the biosynthesized NiO NPs were observed through EDX, which showed three peaks belonging to C, O, and Ni, as shown in Figure 3B. The C element in the sample was from bioactive materials contained in the *S. littoralis Hassk.* extract (Figure 3C). Meanwhile, the Ni (Figure 3D) and O (Figure 3E) elements in the sample proved the success of the synthesis of NiO NPs. NiO NPs are p-type semiconductor materials, with a band gap energy in the range of 3.6–4 eV, and have high conductivity, stable redox kinetics, and efficient charge transport, making them ideal for use as sensing elements [46].

### 3.2. Characterization of Modified Electrodes

**Figure 4:**
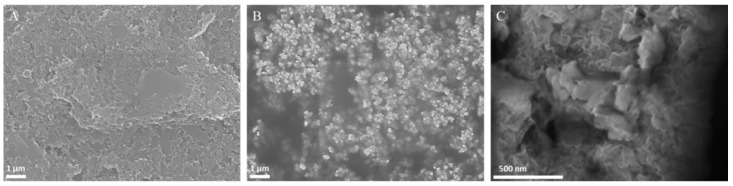
SEM image of (**A**) bare SPE (magnified 10,000×), (**B**) BDD NPs/SPE (magnified 10,000×), and (**C**) NiO NPs/BDD NPs/SPE (magnified 70,000×).

The topography of the SPEs modified with BDD NPs and NiO NPs/BDD NPs was observed by SEM. The bare SPE had a flat but slightly rough and porous surface structure (Figure 4A), while the BDD NP-modified SPE showed a working electrode surface that was covered by polycrystalline BDD NPs measuring <250 nm (Figure 4B). The modification of NiO NPs on the surface of BDD NPs/SPE is shown in Figure 4C, where the distribution of NiO NPs on BDD NPs/SPE indicates that the modification was successful.

### 3.3. Electrochemical Behavior of Ciprofloxacin and Signal-to-Background (S/B) Ratio

**Figure 5:**
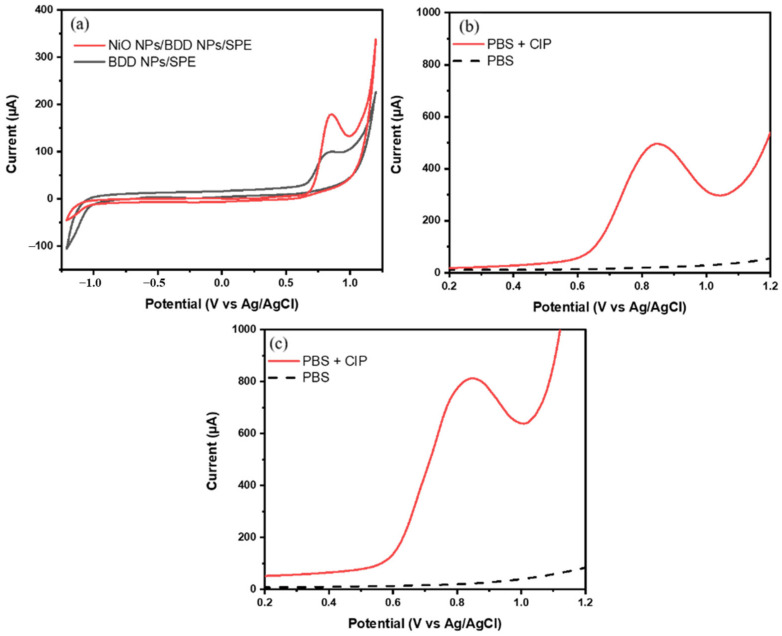
(**a**) CV voltammograms of 100 µM CIP in 0.1 M phosphate buffer solution (pH 7.0) at BDD NPs/SPE and NiO NPs/BDD NPs/SPE and SWV voltammograms of CIP S/B measurement at BDD NPs/SPE (**b**) and NiO NPs/BDD NPs/SPE (**c**).

The electrochemical responses of CIP on BDD NPs/SPE and NiO NPs/BDD NPs/SPE were investigated using CV at a pH of 7.0, which is representative of the pH conditions of real sample matrices such as human urine and milk. Figure 5a shows the cyclic voltammetry responses of 100 μM CIP in 0.1 M phosphate buffer solution (pH 7.0). The CV voltammograms show the presence of an anodic peak of CIP at around 0.85 V. Compared with NiO NPs/BDD NPs/SPE, the peak current of CIP on BDD NPs/SPE was smaller, which proved that the electrode modified with NiO NPs had better electrical conductivity for the detection of CIP.

Determination of the signal-to-background (S/B) ratio was carried out to investigate the level of sensitivity of the sensor. Figure 5b and Figure 5c show the SWV voltammograms of 0.1 M phosphate buffer solution (pH 7.0) measured using BDD NPs/SPE and NiO NPs/BDD NPs/SPE, respectively. The S/B ratios obtained were 23 and 36 for BDD NPs/SPE and NiO NPs/BDD NPs/SPE, respectively. The combination of BDD NPs and NiO NPs resulted in a significant increase in the S/B ratio, which was achieved through a decrease in the background current and an increase in the CIP anodic peak current. Therefore, it can be concluded that the modification of the SPE with BDD NPs and NiO NPs was successful.

**Figure 6:**
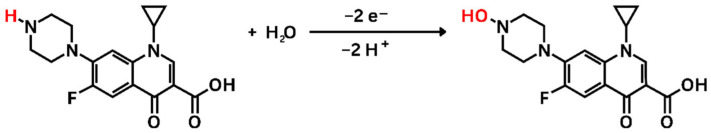
Plausible electrochemical oxidation reaction of CIP.

The oxidation reaction of CIP involves an even number of electrons and protons. The CIP structure consisted of a secondary amine group (-NH) and a primary center structure, with a lone pair of electrons that acted as electron donors. The only active site that can be oxidized is the secondary amine group. The oxidation reaction of the -NH group produces an N-hydroxylation derivative (-N-OH) through the loss of two protons and two electrons, as shown in Figure 6 [47].

**Figure 7:**
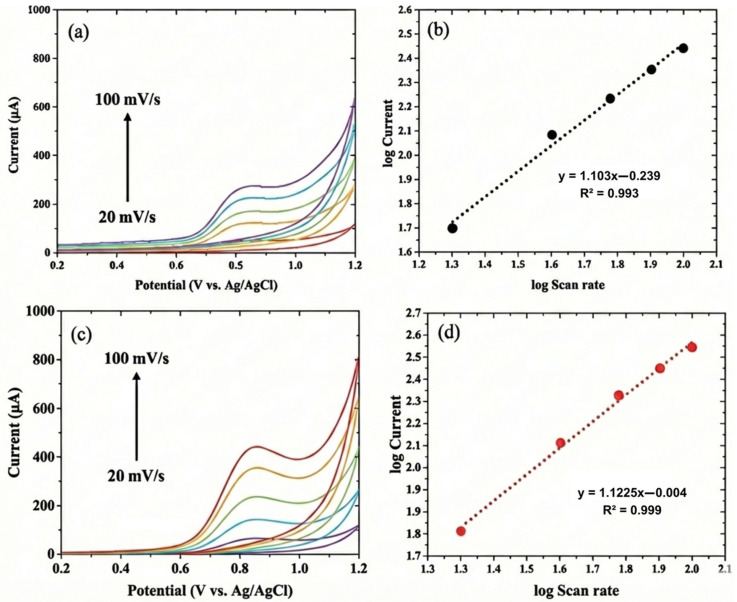
Cyclic voltammograms of 100 µM CIP in 0.1 M phosphate buffer solution (pH 7.0) at different scan rates (20, 40, 60, 80, 100 mV/s) and a plot of log scan rate vs. log current (**a**,**b**) at BDD NPs/SPE and (**c**,**d**) at NiO NPs/BDD NPs/SPE.

The effect of scan rate was studied using CV on 100 µM CIP in 0.1 M phosphate buffer solution (pH 7.0) with scan rate variations of 20, 40, 60, 80, and 100 mV/s. From Figure 7a and Figure 7c, it can be seen that increasing the scan rate results in a linear increase in the anodic peak current of CIP, with linear regression equations $I_{p}\left(\mu A\right)=40.315v-133.829\;(R^{2}=0.995)$ and $I_{p}\;\left(\mu A\right)=68.540v-255.208\;(R^{2}=0.991)$ for BDD NPs/SPE and NiO NPs/BDD NPs/SPE, respectively. Figure 7b and Figure 7d show that the linear regression of the plot of $\log I_{p}vs.\log v$ obtained linear regression equations $\log I_{p}=1.103\log v+0.239\;(R^{2}=0.993)$ and $\log I_{p}=1.225\log v+0.004\;(R^{2}=0.999)$ for BDD NPs/SPE and NiO NPs/BDD NPs/SPE, respectively. From the slope values, it can be concluded that the CIP detection electrode process is controlled by the adsorption process.

### 3.4. Electrochemical Detection of CIP

**Figure 8:**
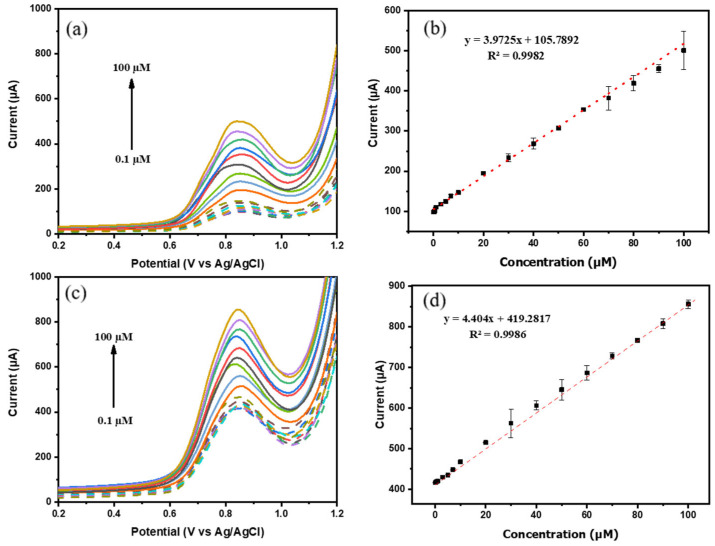
SWV voltammograms of CIP in different concentrations and calibration curve (**a**,**b**) at BDD NPs/SPE and (**c**,**d**) NiO NPs/BDD NPs/SPE.

The ability of the electrode to detect CIP was carried out under a related pH of real samples (urine, milk). Since this study aimed to demonstrate the feasibility of performance enhancement on BDD-based SPEs using green-synthesized NiO NPs, systematic evaluation of pH effects was not undertaken. Under the selected near-neutral conditions, the sensor exhibited stable and well-defined CIP responses suitable for practical analysis. The SWV measurements were performed at varying CIP concentrations in the range of 0.1–100 µM. As shown in Figure 8a, the anodic peak current increased proportionally with increasing CIP concentration. The calibration curve demonstrates a linear correlation between peak current and CIP concentration, described by the regression equations $I_{p}\;\left(\mu A\right)=3.973C+105.789\;(R^{2}=0.998)$ and $I_{p}\;\left(\mu A\right)=4.404C+419.282\;(R^{2}=0.999)$ for BDD NPs/SPE and NiO NPs/BDD NPs/SPE, respectively.

The LOD and limit of quantification (LOQ) were calculated using Equations (1) and (2), respectively, where σ was the standard deviation of the blank (*n* = 3) and *m* was the slope obtained from the regression equation.

(1) $$\mathrm{LOD}=3\frac{\sigma }{m}$$

(2) $$\mathrm{LOQ}=10\frac{\sigma }{m}$$

The BDD NPs/SPE electrode resulted in LOD and LOQ values of 0.109 and 0.365 µM, while the NiO NPs/BDD NPs/SPE electrode resulted in 0.054 and 0.182 µM. Table 1 shows a comparative study of CIP detection methods on different electrodes and voltammetry techniques. The proposed NiO NPs/BDD NPs/SPE demonstrated superior practical performance. Relative to GO/SPE and rGO–SnO_2_/SPE sensors, the present electrode achieved significantly higher sensitivity with lower background while maintaining an equally simple drop-casting method. Unlike molecularly imprinted-based sensors that require multistep electrode modification and imprinting polymerization, the present approach integrates green-synthesized NiO NPs with BDD nanostructures through a simple drop-casting process, with reliable selectivity. CRGO/GCE sensors require an additional electrode polishing step before the modification. The proposed design offers significantly higher sensitivity while providing a more compact system based on SPEs. Compared with PBE, the proposed sensor achieved higher sensitivity, making it more suitable for trace CIP analysis.

**Figure 9:**
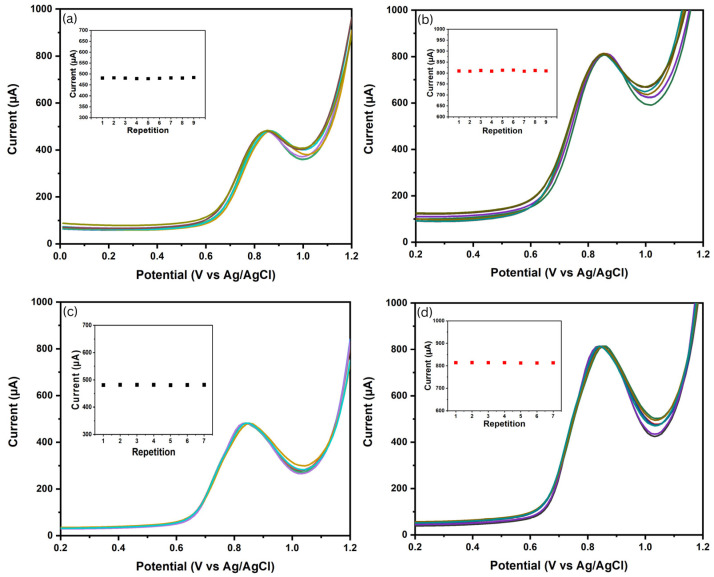
SWV voltammogram demonstrating reproducibility with inset graphs presenting the current values as a function of repeated measurement: (**a**) BDD NPs/SPE and (**b**) NiO NPs/BDD NPs/SPE. While SWV voltammogram for repeatability measurement shown in: (**c**) BDD NPs/SPE and (**d**) NiO NPs/BDD NPs/SPE.

The precision of BDD NPs/SPE and NiO NPs/BDD NPs/SPE were evaluated through reproducibility and repeatability tests, where an electrode could be considered reliable when the RSD values were <2% [53]. Reproducibility testing performed over nine consecutive days with different electrodes demonstrated stable anodic peak currents (Figure 9a,b), with average values of 489.945 ± 1.676 μA (RSD = 0.348%) and 810.465 ± 2.003 μA (RSD = 0.247%) for BDD NPs/SPE and NiO NPs/BDD NPs/SPE, respectively. The results of the experiment indicated that both electrodes showed excellent precision and accuracy. Furthermore, repeatability testing, conducted through seven consecutive measurements using the same electrode on the same day, showed stable CIP anodic peak currents without significant changes in current intensity or potential shift (Figure 9c,d). The average peak currents were found to be 481.366±0.534 μA (RSD=0.111%) and 813.328±0.871 μA(RSD=0.107%) for BDD NPs/SPE and NiO NPs/BDD NPs/SPE, respectively.

**Figure 10:**
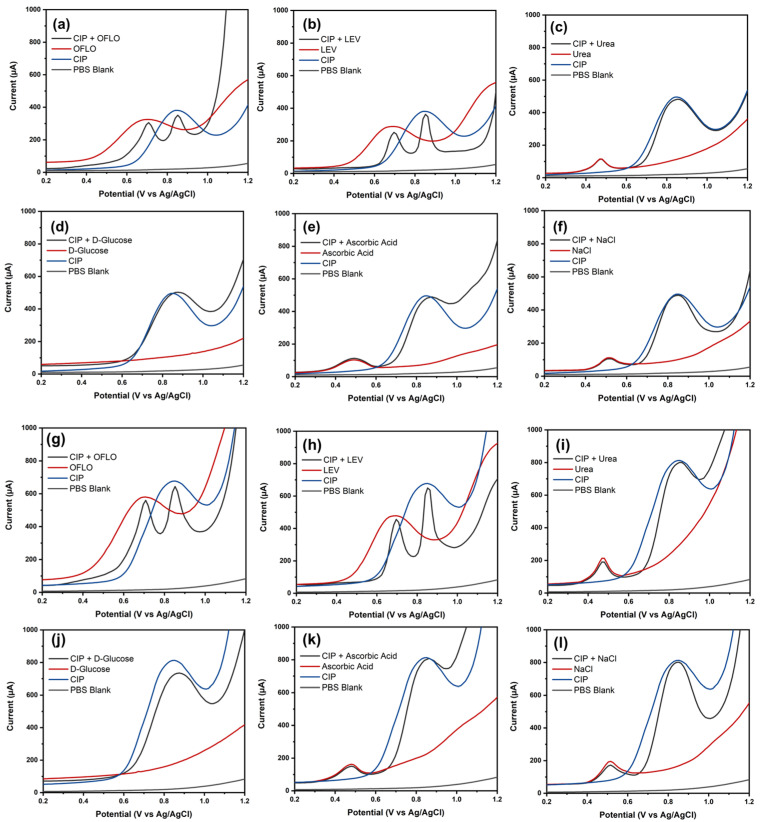
SWV voltammogram of selectivity measurement with the presence of 60 µM OFLO, 60 µM LEV, and 10 µM urea, 100 µM D-glucose, 60 µM ascorbic acid, and 4 µM NaCl (**a**–**f**) at BDD NPs/SPE and (**g**–**l**) at NiO NPs/BDD NPs/SPE.

The ability of electrochemical sensors to distinguish target analytes from interfering substances is an important aspect. To investigate the accuracy of the sensor in measuring the response of analytes in the presence of impurities or interfering molecules, BDD NPs/SPE and NiO NPs/BDD NPs/SPE were used to measure CIP in the presence of six interfering substances, including D-glucose, LEV, OFLO, ascorbic acid, NaCl, and urea. Figure 10a–f and Figure 10g–l show the selectivity measurement of BDD NPs/SPE and NiO NPs/BDD NPs/SPE, with the presence of various interfering compounds, respectively. With the presence of urea, ascorbic acid, and NaCl, an anodic peak appeared around +0.5 V, resulting from interference oxidation, such as ascorbic acid, especially the enediol structure into dehydroascorbic acid [54]. Similarly, urea oxidation into N_2_ could also be possible in the SWV response [55]. However, because NaCl must not produce a peak in the said region, and the peaks are at a similar position compared to urea and ascorbic acid, there is a possibility that the peaks in all three interferences originate from electrode impurities [56]. The measurement showed that both electrodes could distinguish the target analyte with minimal discrepancies in the presence of an interfering compound.

### 3.5. Real Sample Measurement

**Figure 11:**
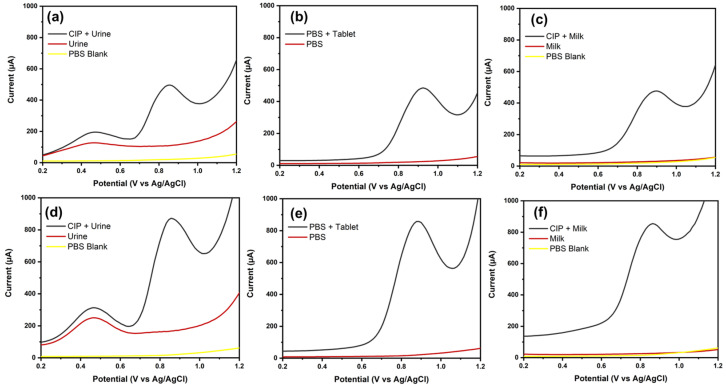
SWV voltammogram real sample measurement of 100 µM CIP in spiked human urine, spiked cow’s milk, and commercial tablets dissolved in PBS (**a**–**c**) at BDD NPs/SPE and (**d**–**f**) NiO NPs/BDD NPs/SPE.

To evaluate the capability of the proposed electrochemical sensor, CIP detection was carried out in human urine, commercial tablets, and cow’s milk. Detection was conducted using the SWV electrochemical technique on tablets that had been dissolved and diluted to a concentration of 100 µM and cow’s milk and human urine that were spiked with 100 µM CIP in 0.1 M phosphate buffer solution (pH 7.0). Figure 11a–c and Figure 11d–f present the real sample measurement result for human urine, commercial tablets, and cow’s milk, carried out by BDD NPs/SPE and NiO NPs/BDD NPs/SPE, respectively. The measurement results showed satisfactory CIP recovery rates for both BDD NPs/SPE and NiO NPs/BDD NPs/SPE (Table 2). Therefore, the proposed method proved to be effective in detecting CIP and has high potential to be further developed for application in various real samples.

## 4. Conclusions

In conclusion, a novel electrochemical sensor is demonstrated based on BDD NP- and NiO NPs/BDD NP-modified SPEs for the sensitive determination of CIP. The fabricated sensor was successfully used to detect CIP in commercial tablets, milk, and human urine with no significant interference from possible interfering substances. Furthermore, the sensor exhibited high sensitivity, low detection limit, good precision and reproducibility, and long-term stability. The proposed method is easy and simple to fabricate, has a fast response, and is low-cost compared to other analytical techniques used to detect CIP. Based on the performance of the fabricated electrode, it has potential for therapeutic drug monitoring of CIP in several real samples.

## Figures and Tables

**Table 1:** Performance comparison for CIP detection from other works.

Electrode	Analytical Methods	Detection Limit (µM)	Linear Range (µM)	Reference
GO/SPE	DPSV	0.30	1–8	[48]
rGO-SnO2/SPE	SWV	2.03	30–100	[16]
Ch-AuMIP/GCE	DPV	0.21	1–100	[49]
PBE	DPV	4.96	9.9–220	[50]
CRGO/GCE	SWV	0.21	6–40	[51]
MIP/GCE	DPV	11.2	50–5000	[52]
BDD NPs/SPE	SWV	0.109	0–100	This work
NiO NPs/BDD NPs/SPE	SWV	0.054		

**Table 2:** Comparison of CIP detection in a real sample on BDD NPs/SPE and NiO NPs/BDD NPs/SPE.

Electrode	Sample	Spiked (µM)	Found (µM)	% Recovery
BDD NPs/SPE	Tablet	100	96.50	96.50%
	Milk	100	95.13	95.13%
	Human urine	100	98.61	98.61%
NiO NPs/BDD NPs/SPE	Tablet	100	99.39	99.39%
	Milk	100	98.11	98.11%
	Human urine	100	99.59	99.59%

## Data Availability

The data presented in this research are available from the corresponding author.

## Abbreviations

The following abbreviations are used in this manuscript:

CIP	Ciprofloxacin
NiO NPs	Nickel oxide nanoparticles
BDD NPs	Boron-doped diamond nanoparticles
LEV	Levofloxacin
OFLOJ	Ofloxacin
SPE	Screen-printed electrode
SWV	Square-wave voltammetry
CV	Cyclic voltammetry
rGO	Reduced graphene oxide
H_2_SO_4_	Sulfuric acid
Na_2_HPO_4_	Disodium hydrogen phosphate
NaH_2_PO_4_	Sodium dihydrogen phosphate
NaCl	Sodium chloride
GCE	Glassy carbon electrode
